# Giant ankyrin-G regulates cardiac function

**DOI:** 10.1016/j.jbc.2021.100507

**Published:** 2021-03-04

**Authors:** Omer Cavus, Jordan Williams, Hassan Musa, Mona El Refaey, Dan Gratz, Rebecca Shaheen, Neill A. Schwieterman, Sara Koenig, Steve Antwi-Boasiako, Lindsay J. Young, Xianyao Xu, Mei Han, Loren E. Wold, Thomas J. Hund, Peter J. Mohler, Elisa A. Bradley

**Affiliations:** 1 Department of Physiology and Cell Biology, The Ohio State University, Columbus, Ohio, USA; 2 The Frick Center for Heart Failure and Arrhythmia, Dorothy M. Davis Heart and Lung Research Institute, The Ohio State University, Columbus, Ohio, USA; 3 Department of Biomedical Engineering, College of Engineering, The Ohio State University, Columbus, Ohio, USA; 4 Department of Internal Medicine/Division of Cardiovascular Medicine, The Ohio State University, Columbus, Ohio, USA

**Keywords:** ankyrin-G, giant ankyrin-G, Nav1.5, arrhythmia, cytoskeleton, cardiovascular disease, myocytes, animal model, AIS, axonal initial segment, AnkG, ankyrin G, CVD, cardiovascular disease, DAD, delayed afterdepolarizations, DCM, dilated cardiomyopathy, EAD, early afterdepolarizations, HFP, high-frequency pacing, VGSC, voltage-gated sodium channels

## Abstract

Cardiovascular disease (CVD) remains the most common cause of adult morbidity and mortality in developed nations. As a result, predisposition for CVD is increasingly important to understand. Ankyrins are intracellular proteins required for the maintenance of membrane domains. Canonical ankyrin-G (AnkG) has been shown to be vital for normal cardiac function, specifically cardiac excitability, *via* targeting and regulation of the cardiac voltage-gated sodium channel. Noncanonical (giant) AnkG isoforms play a key role in neuronal membrane biogenesis and excitability, with evidence for human neurologic disease when aberrant. However, the role of giant AnkG in cardiovascular tissue has yet to be explored. Here, we identify giant AnkG in the myocardium and identify that it is enriched in 1-week-old mice. Using a new mouse model lacking giant AnkG expression in myocytes, we identify that young mice displayed a dilated cardiomyopathy phenotype with aberrant electrical conduction and enhanced arrhythmogenicity. Structural and electrical dysfunction occurred at 1 week of age, when giant AnkG was highly expressed and did not appreciably change in adulthood until advanced age. At a cellular level, loss of giant AnkG results in delayed and early afterdepolarizations. However, surprisingly, giant AnkG cKO myocytes display normal *I*_*Na*_, but abnormal myocyte contractility, suggesting unique roles of the large isoform in the heart. Finally, transcript analysis provided evidence for unique pathways that may contribute to the structural and electrical findings shown in giant AnkG cKO animals. In summary, we identify a critical role for giant AnkG that adds to the diversity of ankyrin function in the heart.

Cardiovascular disease (CVD) characterized by heart failure (HF) and arrhythmia currently impacts >6 million people in the United States each year, with anticipation of growth to 9 million by 2030 (1, 2). It is therefore, increasingly important to understand the etiology and risk factors associated with the development of CVD. To that end, heritable predisposition to HF and arrhythmia remains a vital area for continued study. More specifically, a focus on unique cell types in the myocardium, such as excitable cells, becomes critically important.

Excitable cell function in the human neurologic and cardiovascular systems relies on complex interactions between ion channels and structural proteins; ankyrins are one such membrane-associated protein. More specifically, ankyrin-G (AnkG) has been investigated first in neurologic tissue and later in the heart. The function of canonical AnkG (220 kDa) in neurologic tissue is well described, where it is important for regulating membrane and intracellular protein complexes necessary for normal neurologic function (3, 4) In the heart, our group has shown that canonical AnkG is necessary for Na_v_ channel (Nav1.5) targeting, and in concert with βIV spectrin and CaMKII, for Nav1.5 membrane regulation (5, 6, 7, 8, 9) More recently, we have shown that canonical AnkG has been linked to cardiac remodeling and established as a cause for the development and progression of human HF (9), highlighting that ankyrins may be critical for both structural and electrophysiologic function.

Both *ANK2* and *ANK3* encode in canonical and “giant” isoforms. In neurologic tissue, “giant” AnkG (∼400 kDa) in particular has been studied and found to function in conductance and electrical signaling. At the axonal initial segment (AIS), giant AnkG operates as a membrane-associated adaptor protein that has been implicated in organizing voltage-gated sodium channels (Nav), βIV spectrin, KCNQ2/3, and neurofascin (10, 11). Although our knowledge about giant AnkG function in neurologic tissue continues to evolve, it has remained unclear whether giant AnkG is present in the heart and how it behaves in relation to cardiac structure and electrical function. Therefore, this highlights a gap in our current understanding of roles for the giant AnkG isoform in cardiovascular function and in CVD.

In the present manuscript, we have identified giant AnkG within the heart of both young (1 week old) and adult-aged animals (22 weeks old). To define the role of giant AnkG in the heart, we created a new model of giant AnkG deficiency in postnatal myocytes (αMHC-Cre). Mice deficient in giant AnkG display a dilated left ventricle, gross posterior wall thinning, and systolic dysfunction, characteristic of a dilated cardiomyopathy (DCM) phenotype in the neonatal period. In addition, these mice exhibit electrical dysfunction including ventricular arrhythmia and high-degree (third-degree and complete) heart block. Notably, unlike canonical AnkG, these phenotypes appear to be independent of Na_V_1.5 channel function and instead appear linked with aberrant Ca^2+^ handling. Collectively, our data support that a single ankyrin gene produces at least two separate products, canonical AnkG and giant AnkG, each with unique roles in cardiac structure and electrical function, the latter of which acts *via* a novel sodium-channel-independent mechanism in the developing myocardium.

## Results

### Giant ankyrin-G is enriched in the heart of young animals

**Figure 1:**
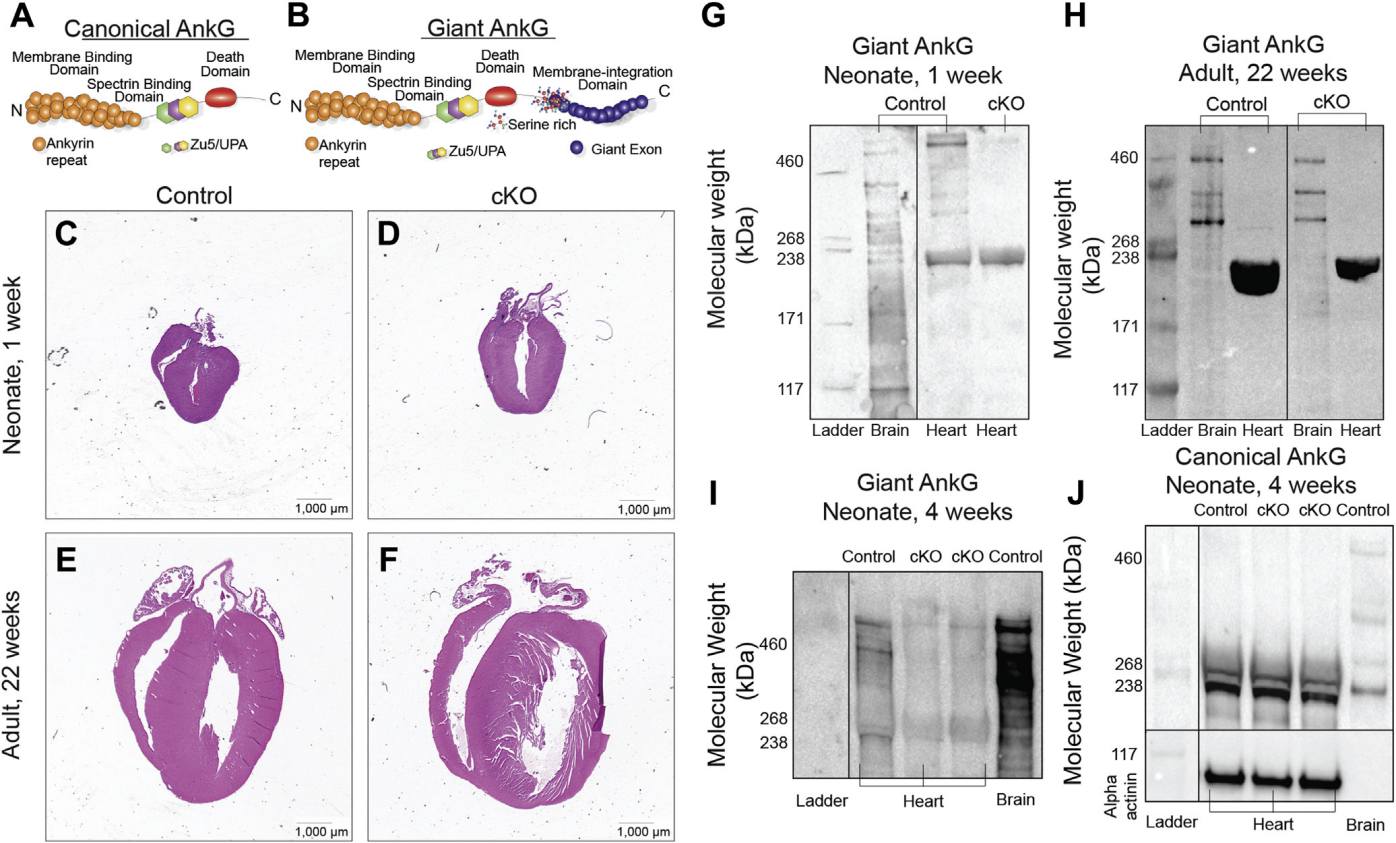
**Giant Ank-G is enriched in young animals.***A* and *B*, domain organization of canonical *versus* giant AnkG. *A*, the “giant” isoform includes not only the membrane-binding and spectrin-binding domains, but also a membrane-integration domain (*B*). Giant AnkG cKO animals demonstrated mild chamber dilatation and ventricular thinning, supportive of eccentric hypertrophy at both 1 and 22 weeks of life (*C*–*F*). In 1-week control animals, giant AnkG is highly expressed in the myocardium and brain, yet barely detectable in the cKO animal, confirming selective cardiac silencing (*G*); however giant AnkG is functionally gone in both control and cKO adult (22 weeks) animal heart, but not brain tissue (*H*). In 4-week-old floxed and cKO mice, giant AnkG is highly expressed in the myocardium and brain, yet nearly absent in the cKO animal (*I*); however, canonical AnkG remains expressed in control and cKO animals at the same age (normalized to alpha-actinin, *J*). In *G*–*J*, heart sample analyses was performed on same gel, nonadjacent lanes.

Canonical AnkG (*ANK3* in human, *Ank2* in mice, 190 kD, Fig. 1*A*) is essential for normal ion channel targeting in neurologic tissue (12) and is important both *in vitro* (7, 13) and *in vivo* in the heart (6, 9). Similarly, giant AnkG isoforms (480 kD and 270 kDa, Fig. 1*B*) have been identified in neurologic tissue, where they function in the assembly of the AIS and nodes of Ranvier (10). However, it is unknown whether giant AnkG is present and/or functions in cardiovascular tissue. Although abnormal expression of canonical AnkG has been demonstrated in both ischemic and traumatic neurologic models (14, 15), as well as ischemic HF (9), the presence and role of giant AnkG in cardiovascular tissue are not described. In fact, prior work from Jenkins *et al.* (10) suggests that giant AnkG is not present in heart and/or expressed at very low levels.

To both directly investigate the expression of giant AnkG in the heart (requires robust negative control) and test a potential *in vivo* role of giant AnkG in heart, we generated a mouse line with cardiac-restricted giant ankyrin-G knockout utilizing techniques described (10). The MHC-Cre mice provide myocyte-specific elimination during the first week of life (10). We studied male and female *Ank3*^f/f^ control (flox) mice and cKO mice in the neonatal period (day of life 7) and adulthood (22 weeks of age).

Giant AnkG cKO mice were viable and displayed no differences in size, weight, feeding, or grooming. Similar to mice with selective giant AnkG deletion in the brain, cardiac-restricted giant AnkG cKO mice survive neonatal life and demonstrate no gross motor abnormalities (10). Giant AnkG cKO animals (neonate and adult) displayed mild left ventricular (LV) dilatation and LV thinning (Fig. 1, *C*–*F*) consistent with eccentric hypertrophy. Notably, immunoblots illustrate that giant AnkG is easily detected in heart lysate of mice at 1 week of age (brain utilized as positive control, Fig. 1, *G* and *H*). This expression in young hearts is significantly reduced in cKO; absolute cKO heart loss of giant AnkG is likely due to incomplete activity of the αMHC promoter during this time of development and/or giant AnkG expression in nonmyocytes. Further, the expression of giant AnkG was significantly abolished in the cKO mice compared with the floxed littermates at 4 weeks of age, while canonical AnkG protein expression did not vary between the different genotypes (Fig. 1, *I* and *J*). Relative expression of the large isoform (qPCR) confirmed near absence of giant AnkG in the 4-week cKO animals (Fig. S1). However, here we demonstrate for the first time that giant AnkG is expressed in cardiac tissue in early development independent of the robust expression of the canonical AnkG isoform.

In summary, giant AnkG is expressed in the heart, albeit in significantly lower levels than canonical AnkG. Further, AnkG cKO mice display cardiac structural abnormalities.

### Giant AnkG deficiency results in aberrant cardiac function

A primary role of canonical AnkG in the heart has been shown to have occurred through interaction with Na_v_1.5, the primary cardiac voltage-gated Na_v_ channel (5, 6, 7, 8). However, more recent evidence suggests that canonical AnkG also likely functions in cardiac myofilament integration and is necessary for normal compensatory cardiac remodeling (9), yet the role for giant AnkG in cardiac function has not been determined.

**Figure 2:**
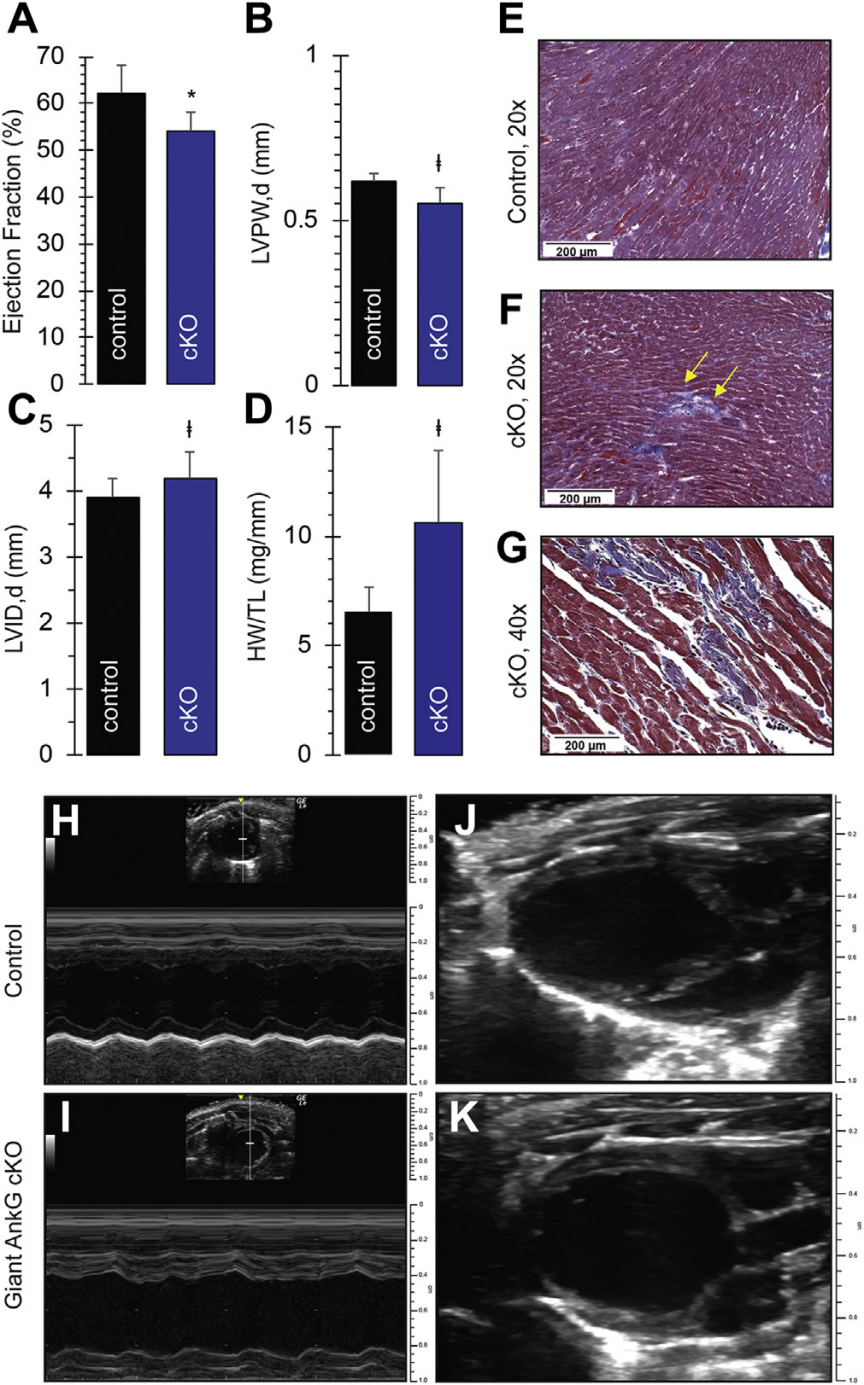
**Giant AnkG cKO mice demonstrate a dilated cardiomyopathy phenotype.** Left ventricular systolic function is reduced in giant AnkG cKO mice (EF 54 ± 4% *versus* 62 ± 6%, *p* < 0.001) (*A*). cKO mice display a dilated cardiomyopathy phenotype, including left ventricular (LV) enlargement (LVID,d 4.2 ± 0.4 *versus* 3.9 ± 0.3 mm, *p* < 0.05) (*C*) and LV thinning (LVPW,d 0.55 ± 0.02 *versus* 0.63 ± 0.02 mm *p* < 0.05) (*B*). cKO mice demonstrate increased heart weight, when normalized to tibial length (10.6 ± 3.3 *versus* 6.5 ± 1.2 mg/mm, *p* < 0.05) (*D*). Trichrome staining of control (*E*, 20×) and cKO (*F* 20×, *G* 40×) hearts redemonstrates important changes in the cKO mice (*E* and *G*) including fibrosis (*yellow arrows*, *F*). M-mode and two-dimensional echocardiographic images display dilatation of the LV chamber, thinning of the LV posterior wall, and reduced systolic function (ejection fraction) at 20 weeks (*p* < 0.05) (Control: *H* and *J*; giant AnkG cKO *I* and *K*). *H*–*K*, ∗*p* < 0.001, ∗∗*p* < 0.05. (Echo: Control n = 14, cKO n = 16; Heart weight/tibial length: Control = 5, cKO n = 4).

We examined gross and histologic specimens to evaluate myocardial structure and function in giant AnkG cKO and control mice (age- (22 weeks) and sex-matched). We observed mild dilatation of the LV chamber (LVID,d 4.2 ± 0.4 *versus* 3.9 ± 0.3 mm, *p* < 0.05) and myocardial thinning (LVPW,d 0.55 ± 0.02 *versus* 0.63 ± 0.02 mm *p* < 0.05) in cKO animals (Fig. 2, *A*–*D*, *H*–*K*). Unlike control mice, cKO animals demonstrated increased heart weight when normalized to tibial length (10.6 ± 3.3 *versus* 6.5 ± 1.2 mg/mm, *p* < 0.05), indicative of the eccentric remodeling expected in DCM (Fig. 2*D*). Finally, we observed myocardial fibrosis in cKO animals, consistent with aberrant remodeling (Fig. 2, *E*–*G*). Taken together, these findings provide evidence for an eccentric hypertrophy phenotype such as DCM, a disease process that requires fibrosis and abnormal remodeling, as necessary substrate for the development of both structural and electrical dysfunction.

**Figure 3:**
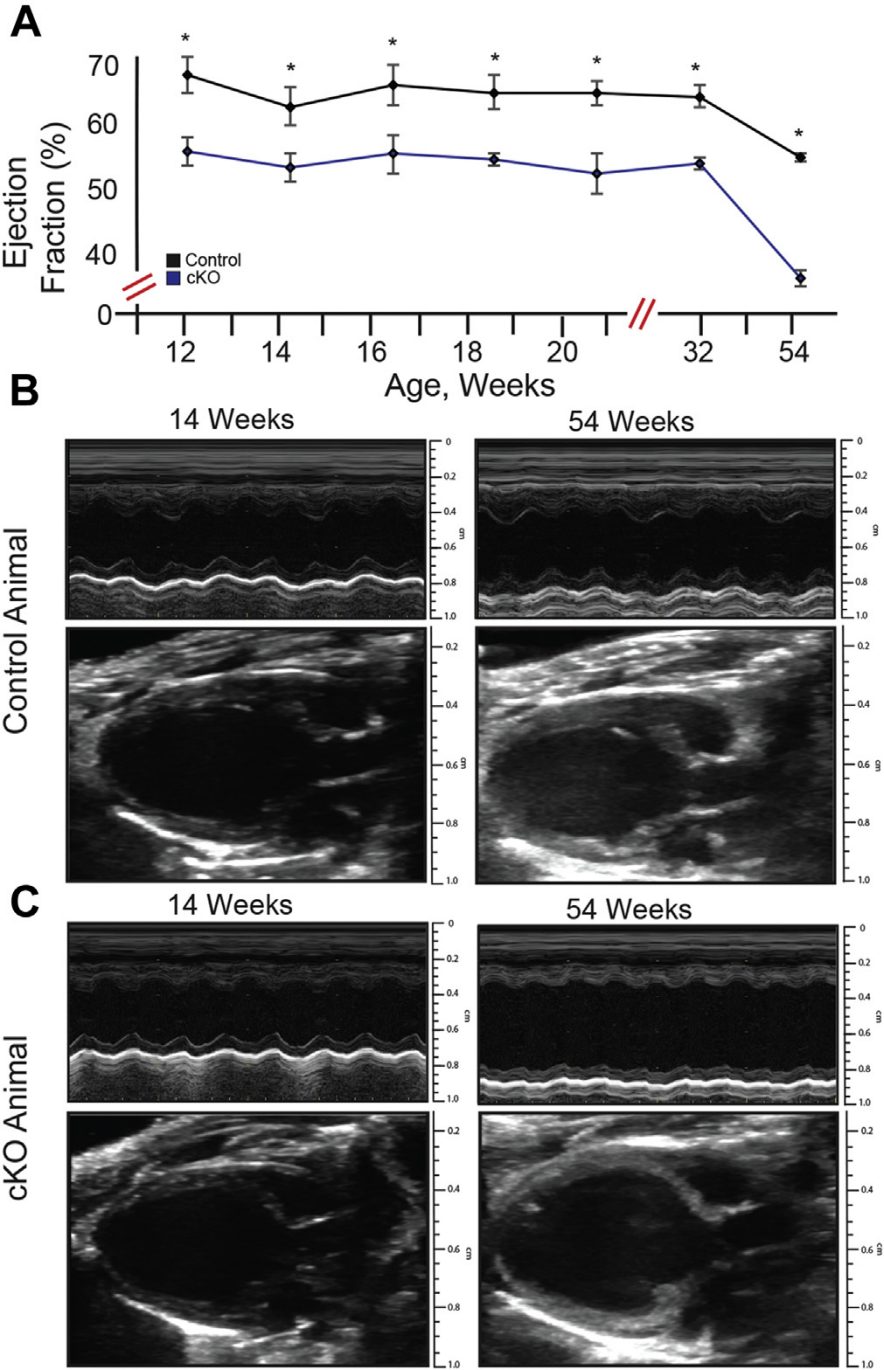
**Giant AnkG is required for normal cardiac structure.** Cardiac structure/function was assessed in young and adult control and cKO mice. For cKO mice, systolic function was significantly lower at each age interval (*A*). Animals evaluated between 12 and 32 weeks did not demonstrate significant overall changes in left ventricular size or function (cKO 12 *versus* 32 weeks: LVID,d 3.9 ± 0.2 *versus* 3.8 ± 0.2 mm, *p* = 0.64, LVPW 0.51 ± 0.10 *versus* 0.50 ± 0.10 mm, *p* = 0.75, EF 56 ± 5 *versus* 54 ± 2%, *p* = 0.41). However, by 54 weeks of age, there was significant dilatation and systolic dysfunction in cKO animals (cKO 12 *versus* 52 weeks: LVID,d 3.9 ± 0.2 *versus* 4.7 ± 30, *p* < 0.01 mm, EF 56 ± 5 *versus* 36 ± 7%, *p* < 0.01) (*A*). Representative M-mode and two-dimensional echocardiography in a Flox-control (*B*) and cKO (*C*) animal are shown. (cKO n = 5, Flox control n = 4) ∗*p* < 0.05.

To investigate the relationship between structural changes and heart function, we performed serial echocardiograms on animals 12 to 32 weeks in age, at 2-week intervals. We also assessed an aged animal cohort (n = 5 cKO, n = 4 control) to determine if there was further impact upon heart structure and late cardiovascular function (54 weeks). Similar to the pathologic data, we observed *in vivo* that cKO animals demonstrated hallmarks of a DCM phenotype, including LV enlargement and thinning (Fig. 3). In concert with the structural changes, we found evidence of LV systolic dysfunction in the cKO animals (ejection fraction/EF 54 ± 4% *versus* 62 ± 6%, *p* < 0.001). Both structure and functional changes were evident at early ages (12 weeks) and did not appreciably change through 32 weeks of age (cKO 12 *versus* 32 weeks: LVID,d 3.9 ± 0.2 *versus* 3.8 ± 0. 2 mm, *p* = 0.64, LVPW 0.51 ± 0.10 *versus* 0.50 ± 0.10 mm, *p* = 0.75, EF 56 ± 5 *versus* 54 ± 2%, *p* = 0.41). However, by 54 weeks of age, LV dilatation and dysfunction significantly worsened in the cKO animals (cKO 12 *versus* 52 weeks: LVID,d 3.9 ± 0.2 *versus* 4.7 ± 0.3 mm, *p* < 0.01, EF 56 ± 5 *versus* 36 ± 7%, *p* < 0.01) (Fig. 3). Thus, giant AnkG is important in early cardiac development and is necessary for normal neonatal heart function. In the absence of giant AnkG, animals displayed eccentric hypertrophy and systolic dysfunction in early life, with further dilatation and systolic dysfunction late in life, implying that although the structural and functional changes occur early, they may also contribute to more significant phenotypes in late adulthood. Alternatively, low levels of giant AnkG in adult mice may be critical for maintaining cardiac structure.

### Giant AnkG supports electrical stabilization of the myocardium during catecholaminergic stress

Canonical AnkG is necessary for cardiac Na_v_1.5 function and recruitment of CaMKIIδ to the intercalated disc. Canonical AnkG loss leads to CaMKII-dependent regulation of *I*_Na,L_ resulting in both conduction defects and arrhythmia (5, 6). Herein, we have shown here that giant AnkG cKO animals demonstrate structural myocardial remodeling and contractile dysfunction (Fig. 2). Given what is known about canonical AnkG in the heart, we hypothesized that the observed structural abnormalities would coexist with electrical dysfunction in the form of ventricular arrhythmias and/or abnormal atrioventricular conduction. Therefore, we tested cardiac-deficient giant AnkG animals to determine if conduction disturbances and arrhythmia were present at rest and/or with catecholamine stimulation.

**Figure 4:**
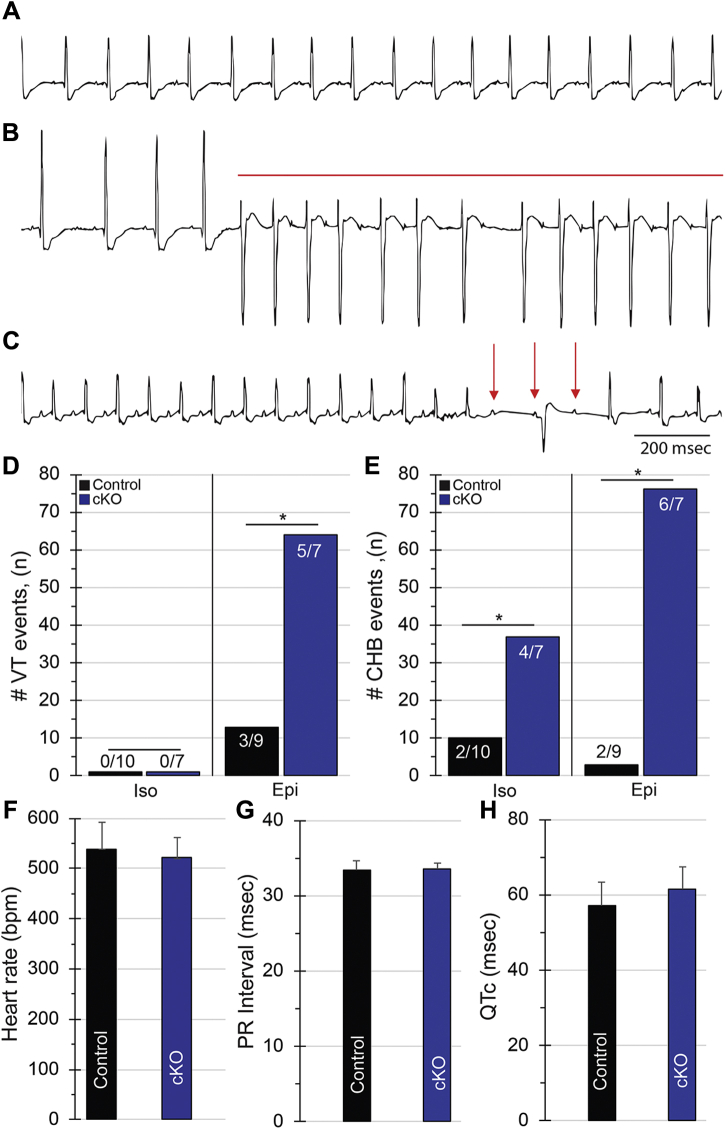
**Giant ankyrin-G cKO mice demonstrate ventricular arrhythmia and high-degree block.** Giant AnkG is required for normal cardiac excitability. On resting telemetry, giant AnkG mice displayed periods of normal sinus rhythm (*A*) with frequent premature ventricular contractions, nonsustained ventricular tachycardia (VT), sustained VT (*red line*, *B*), and complete heart block with atrioventricular dissociation (*red arrows*, *C*), despite having similar resting heart rate, PR, and QTc intervals as compared with control mice (*F*–*H*). With catecholamine exposure (Isoproterenol/Iso (45 min) and Epinephrine/Epi (60 min)), cKO mice displayed significantly more ventricular arrhythmia in response to Epi (13 *versus* 64 events, *p* < 0.0001) (*D*), and more high-degree heart block in response to Iso (10 *versus* 37 events, *p* < 0.0001) and Epi (3 *versus* 76 events, *p* < 0.0001) (*E*) (n/n indicates number of mice affected out of total number of mice studied). ∗*p* < 0.0001. (Control: n = 10, cKO: n = 7).

We performed conscious electrocardiographic monitoring at rest on both cKO and control animals. Electrocardiographic tracings revealed no significant differences in resting ECG parameters (cKO *versus* control; Heart rate (HR): 521 ± 41 *versus* 539 ± 54 bpm, *p* = 0.45, PR Interval: 33 ± 0.8 *versus* 33 ± 1.0 ms, *p* = 0.85, QTc: 59 ± 3 *versus* 63 ± 2 ms, *p* = 0.26) (Fig. S2, Fig. 4, *A*, *F*–*H*). However, with catecholamine exposure, high-degree atrioventricular block was observed more frequently in cKO, *versus* control mice (Iso: 10 *versus* 37 events, *p* < 0.0001; Epi: 3 *versus* 76 events, *p* < 0.0001) (Fig. 4, *C* and *E*). The same animals displayed significantly more ventricular arrhythmia. However, this only occurred in response to epinephrine (13 *versus* 64 events, *p* < 0.0001), as there were no ventricular arrhythmic events in either control or cKO animals in response to the beta-agonist isoproterenol (Fig. 4, *B* and *D*).

### Giant AnkG is required for normal myocyte excitability

**Figure 5:**
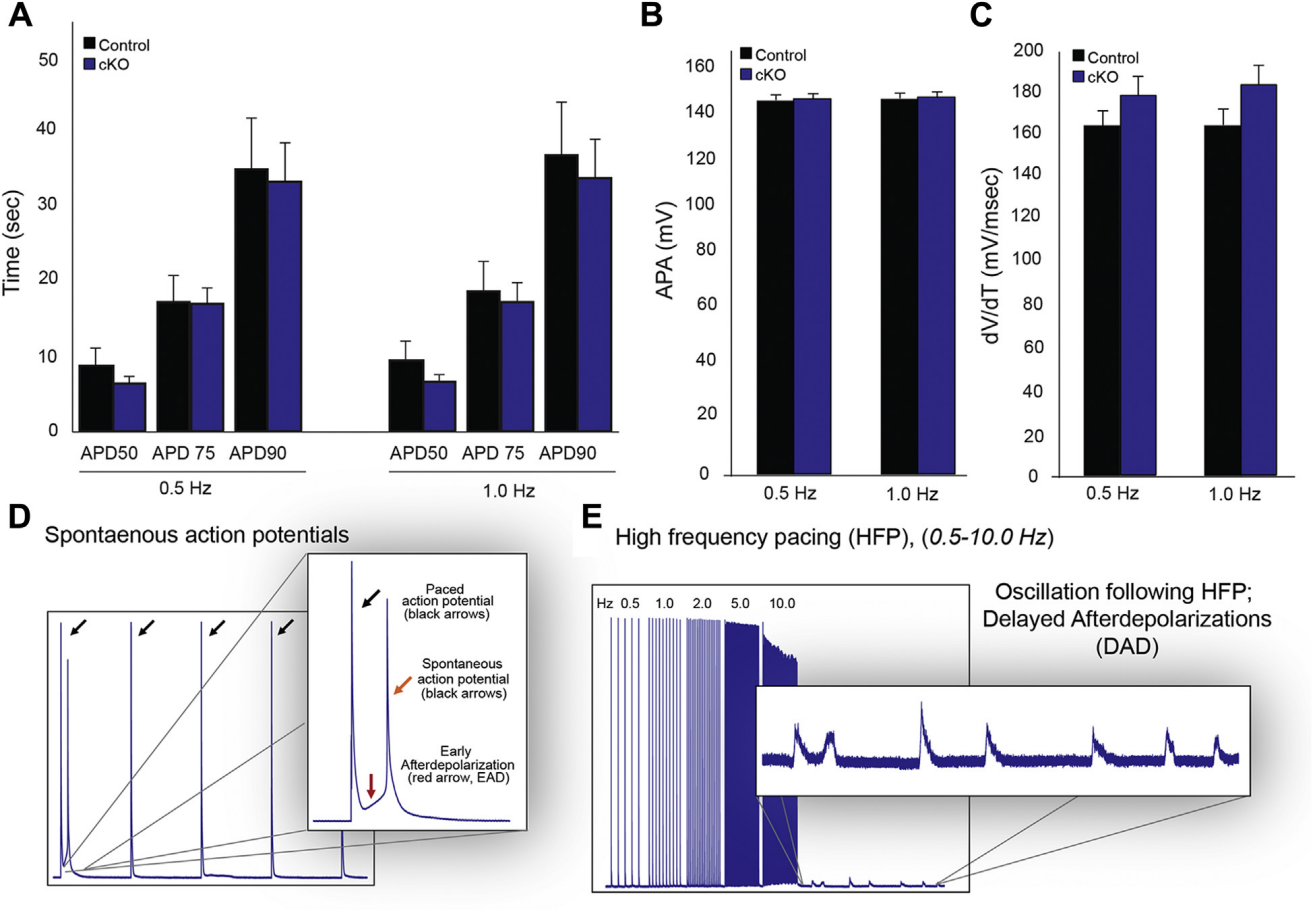
**Spontaneous and pacing-mediated automaticity demonstrated in the absence of giant AnkG.** Action potential profiles in control and cKO animals paced at 0.5 Hz and 1.0 Hz (*A*–*C*). Action potential duration (APD) at 50, 75, and 90% repolarization, action potential amplitude (APA), and the maximum rate of voltage change (dV/dT) are not statistically different between control and cKO ventricular myocytes (*D*). Occasional early afterdepolarizations (*red arrow*) give rise to a spontaneous AP (*orange arrow*), observed in cKO myocytes but not control myocytes (*E*). S1–S5 pacing protocols ranging from 0.5 Hz to 10 Hz initiated a run of delayed after depolarizations (DADs) after cessation of pacing. Control *versus* cKO N = 4,6 and n = 14,18.

To assess the time-dependent transmembrane potential for clues to the cause of electrical dysfunction in giant AnkG cKO animals, (22 weeks), we paced ventricular myocytes at 0.5 and 1.0 Hz and measured action potential duration and amplitude. There was no significant difference in action potential duration (Fig. 5*A*) or amplitude (Fig. 5*B*) at either pacing interval. Similarly, there was no difference in the maximum rate of voltage change (dV/dT) between control and cKO ventricular myocytes (Fig. 5*C*). We next measured myocyte electrical activity in response to specific protocols. During 0.5 Hz pacing, early afterdepolarizations (EADs) were observed in AnkG cKO myocytes and when present, EADs occasionally gave rise to a spontaneous action potential (Fig. 5*D*). Next, cardiac myocytes were subject to a S1-S5 protocol where they were paced at successively higher frequencies ranging from 0.5, 1.0, 2.0, 5.0, and 10.0 Hz for a period of 1 s at each frequency. After cessation pacing, giant AnkG cKO myocytes displayed runs of delayed afterdepolarizations (DADs); these were not observed in control myocytes (Fig. 5*E*). The presence of resting EADs and post-HFP DADs suggests that giant AnkG-deficient myocytes exhibit susceptibility to increased automaticity, likely impacted by a functional role that giant AnkG plays in myocyte stabilization required for normal electrical conduction and cardiac function.

### Giant AnkG functions independent of the primary voltage-gated Na_v_ channel in the heart

**Figure 6:**
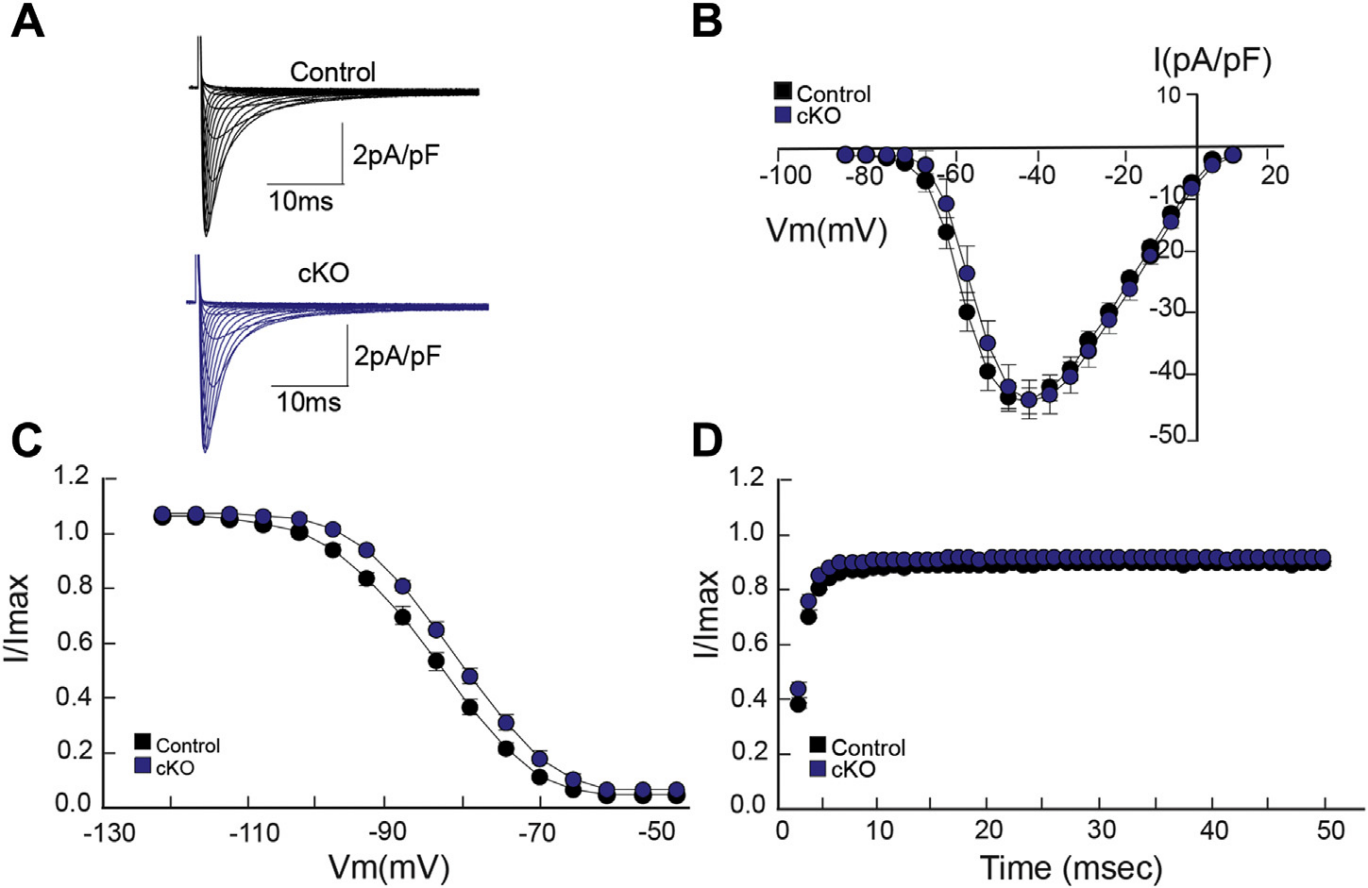
**Giant ankyrin-G-mediated electrical dysfunction is independent of Na**_**v**_**1.5.** Whole-cell recordings from ventricular myocytes evaluating sodium current properties demonstrated no differences in the current voltage relationship (I-V), voltage-dependent inactivation (h’ curve) and time-dependent recovery of *I*_*Na*_ between cKO and control myocytes (*B*–*D*). Representative recordings from control and cKO myocytes are shown in *A*. For *I*_*Na*_ experiments, control *versus* cKO N = 3,3 and n = 13,13.

Canonical AnkG functions in concert with the voltage-gated Na_v_ channel (5). Cardiac disease in AnkG cKO animals has been demonstrated to include increased automaticity, arrhythmogenicity, and moderate- and high-grade conduction abnormalities (6). To determine if giant AnkG functions similar to the canonical isoform, we evaluated sodium channel activity in giant AnkG-deficient animals. We performed cardiomyocyte whole-cell voltage-clamp recordings in ventricular myocytes to evaluate Na_V_1.5-mediated sodium current and found no difference in current density, voltage-dependent inactivation (h’ curve), and time-dependent recovery of *I*_Na_ (Fig. 6, *A*–*D*). The action potential and *I*_*Na*_ findings support that giant AnkG may at least in part function independent of the primary voltage-gated Na_v_ channel in the heart.

**Figure 7:**
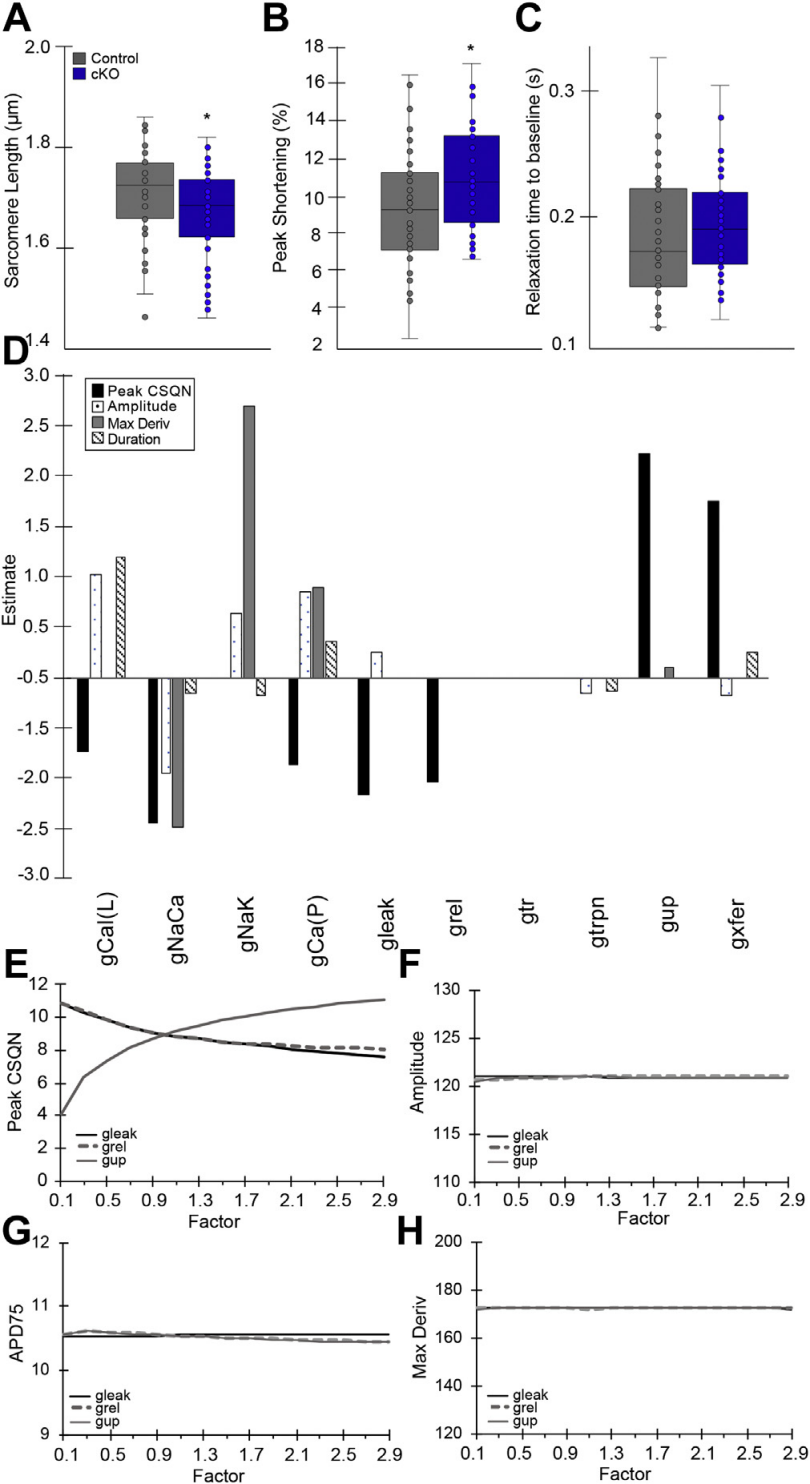
**Cardiomyocyte contraction and computational modeling.** Myocyte sarcomeres from 1-week-old animals were shorter in the cKO group (1.64 ± 0.08 *versus* 1.66 ± 0.11 μm, *p* < 0.05) at baseline (*A*) and demonstrated more robust peak shortening (11.1 ± 2.7 *versus* 9.6 + 3.1%, *p* < 0.05) (*B*) when stimulated, albeit with no change in relaxation time (*C*). Computer simulations to predict mechanism for delayed after depolarizations (DADs) in the absence of overt changes to baseline action potential were performed and peak CSQN was used as a surrogate for DADs. *D*, partial least-squares regression analysis was performed to determine relative impact of major ion channels and transporters in the model on peak CSQN and action potential amplitude, duration at 75% repolarization (APD_75_), and max derivative. A total of 200 single-cell simulations were performed of scaling parameters for the following ion fluxes: L-type Ca^2+^ current (g_Ca(L)_), Na^+^/Ca^2+^ exchanger (g_NaCa_), Na^+^/K^+^ ATPase (g_Nak_), sarcolemmal Ca^2+^ pump (g_Ca(P)_), Ca^2+^ leak flux from the SR (g_leak_), Ca^2+^ release from SR (g_rel_), Ca^2+^ translocation from network to junctional SR (g_tr_), Ca^2+^ flux to troponin (g_trpn_), Ca^2+^ uptake into SR (g_up_), and Ca^2+^ flux from the subspace volume to bulk myoplasm (gxfer). Regression coefficient estimates predict that Ca^2+^ leak, release, and reuptake into SR have a marked effect on peak CSQN with a minimal effect on other action potential properties, and therefore these three scaling factors were then incrementally increased (0.2 steps starting at 0.1–2.9) and evaluated for their effect on (*E*) peak CSQN, (*F*) action potential amplitude, (*G*) APD_75_, and (*H*) max derivative. Ca^2+^ leak and release from the SR had an inversely proportional relationship with peak CSQN, while increasing the maximal Ca^2+^ uptake into SR showed an increase in peak CSQN. *E*, action potential amplitude, APD_75_, and max derivative showed minimal responses to the dramatic changes in SR Ca^2+^ factors (*F*–*H*).

These data, in combination with the AP recordings, including display of abnormal depolarization (both DADs and EADs) in the setting of normal action potential duration and *I*_*Na*_, suggest that giant AnkG may have additional unique functions in the heart. We hypothesized that this could be influenced by calcium dysfunction and therefore analyzed sarcomere length and performed real-time cardiomyocyte functional and calcium studies in neonatal (1 week old) control and cKO animals (n = 4 each group). In the cKO group, myocyte sarcomeres were shorter at baseline (1.64 ± 0.08 *versus* 1.66 ± 0.11 μm, *p* < 0.05) and demonstrated increased percent peak shortening (11.1 ± 2.7 *versus* 9.6 + 3.1%, *p* < 0.05) when stimulated, albeit with no change in relaxation time (Fig. 7, *A*–*C*), consistent with increases in cytosolic Ca^2+^. To further evaluate potential etiologies for abnormal depolarization and slower Ca^2+^ reuptake in the cKO group, we performed computer simulations to predict mechanisms for DADs in the absence of overt changes to baseline action potential. We used the Bondarenko model of the murine ventricular action potential (16), in which DADs are triggered when concentration of Ca^2+^-bound calsequestrin (peak CSQN) in the sarcoplasmic reticulum (SR) reaches 11.5 (mM). The generated regression coefficient estimates predict that Ca^2+^ leak, release, and reuptake into SR have a marked effect on peak CSQN with a minimal effect on other action potential properties (Fig. 7*D*). To further test this hypothesis, these three scaling factors were then incrementally increased over a wide range (0.2 steps starting at 0.1–2.9) and evaluated for their effect on peak CSQN, action potential amplitude, APD_75_, and max derivative. Ca^2+^ leak and release from the SR had an inversely proportional relationship with peak CSQN, while increasing the maximal Ca^2+^ uptake into SR showed an increase in peak CSQN. (Fig. 7*E*) Action potential amplitude, APD_75_, and max derivative showed minimal responses to the dramatic changes in SR Ca^2+^ factors (Fig. 7, *F*–*H*). Together, these data indicate that in the heart, giant AnkG functions independent from the primary voltage-gated Na_v_ channel, with evidence for novel roles in Ca^2+^ metabolism.

### Transcriptional analysis supports an extracellular role for giant ankyrin-G

**Figure 8:**
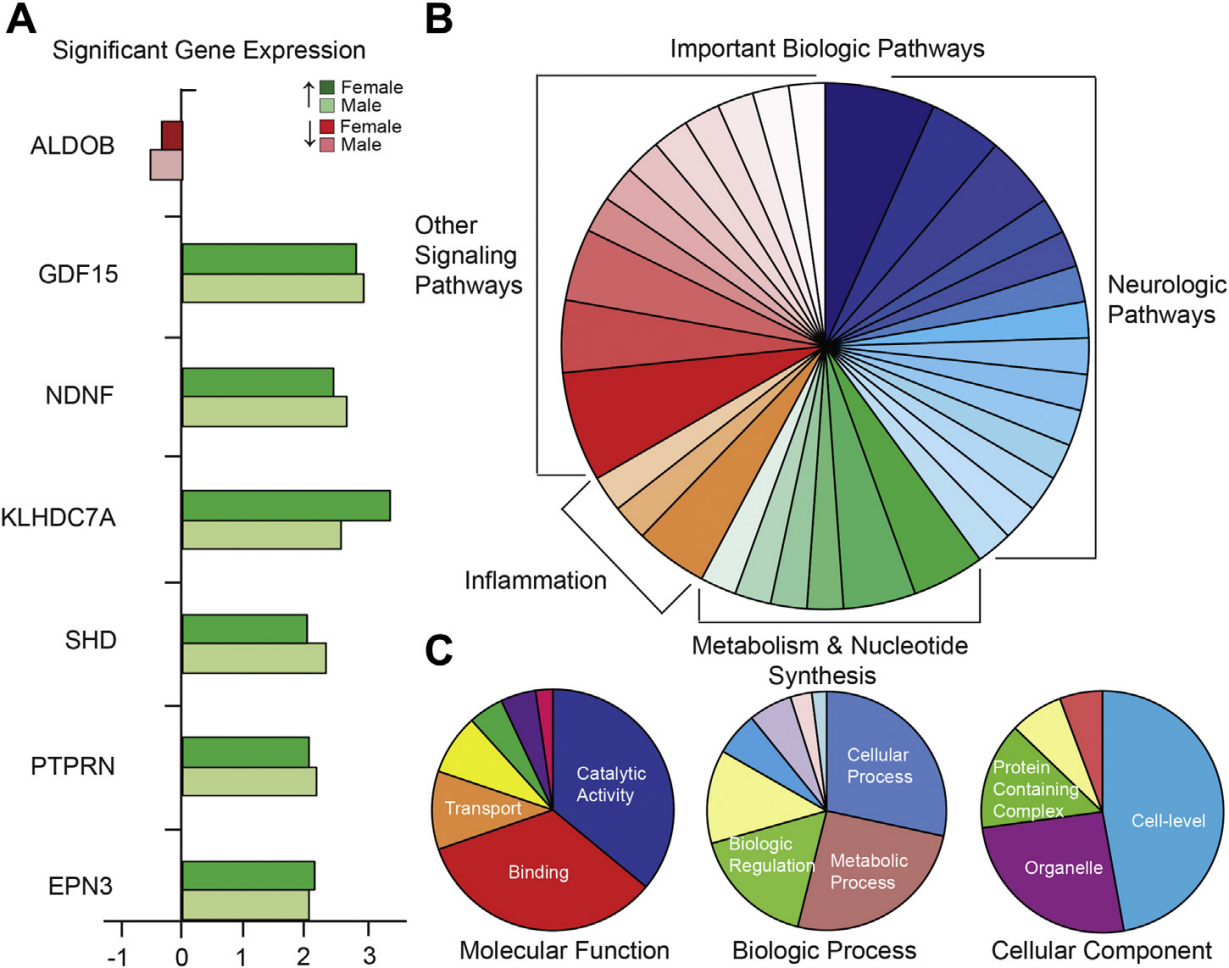
**Transcript analysis.** A total of 12,124 detectable nuclear protein-coding genes were identified as potentially relevant. Data was filtered to include biologic and molecular ontologic analysis of any gene that was significantly different (*p* < 0.05) in both male and female cKO (*versus* flox-control) animals. Any gene with a twofold increase or decrease is shown here in both male and female control and cKO animals (*A*). Relevant biologic and molecular pathways in differentially expressed genes (102 genes, 45 pathways in both male and female animals, *p* < 0.05) are shown (*B* and *C*). Functions/Processes/Components accounting for >10% within each category are labeled in *white* (*C*). The full list of potentially relevant pathways is available in the data supplement.

Given that there is scarce data about giant AnkG function in cardiac tissue, we performed RNA sequencing and transcript analysis of the myocardium in control and cKO male (control n = 3, cKO n = 3) and female (control n = 3, cKO n = 3) animals. In part, we aimed to determine if unique pathways might explain the phenotype we observed, in the presence of normal action potential and *I*_*Na*_. In the heart, there were 12,124 total transcripts analyzed. Of these, there were 113 coding transcripts that were either significantly either increased (≥1.5-fold, *p* < 0.05) or decreased (≤1.5-fold, *p* < 0.05) in cKO animals irrespective of sex and seven coding transcripts that were significantly increased in *both* male and female cKO animals (no coding transcripts were ≥1.5-fold decreased in *both* male and female cKO animals) (Fig. 8*A*). Of the seven genes upregulated, four were found to be potentially relevant in CVD and included: Epsin 3 (*EPN3*) (17), growth differentiation factor 15 (*GDF15*), neuron-derived neurotrophic factor (*NDNF*) (18), and protein tyrosine phosphatase receptor type N (*PTPRN*) (Table 1).

**Table 1:** Upregulated key transcripts implicated in giant AnkG-mediated pathways

Gene	Protein nomenclature	Implication in cardiovascular disease
*Epn3*	Epsin 3	Deficiency in Epsin impairs endocytosis by stalling actin-dependent invagination of clathrin coated pits (17).
*Gdf15*	Growth differentiation factor 15	Encodes ligand for TGF-β, which activates SMAD family transcription factors that regulate gene expression. Expressed in location of cell injury, hypoxia, and inflammation.
*Klhdc7a*	Kelch domain containing 7A	No data in cardiovascular disease
*Ndnf*	Neuron derived neurotrophic factor	Proangiogenic and upregulated in ischemic skeletal and cardiac models (18).
*Ptprn*	Protein tyrosine phosphatase receptor type N	Member of protein tyrosine phosphatase family (PTP) that functions as signaling molecule to regulate growth, differentiation, mitotic cycle, and oncogenic transformation.
*Shd*	Src homology 2 domain containing transforming protein D	No data in cardiovascular disease

To identify potential molecular mechanisms responsible for giant AnkG-mediated cardiovascular disease, we performed gene ontologic analysis (19, 20). We identified and analyzed genes differentially expressed (n = 102, *p* < 0.05) in both male and female cKO animals (21). We then organized these genes into categories that represented important biologic pathways, molecular function, biologic process, and cellular components (Fig. 8, *B* and *C* and Table 2) and found that differentially expressed genes were predominantly important in producing proteins that have previously been described in neurologic pathways (Table 3).

**Table 2:** Gene ontology analysis

Molecular function *(102 genes, 82 hits)*	Biologic process *(102 genes, 102 hits)*	Cellular component *(102 genes, 70 hits)*
Catalytic activity	Cellular process	Cell
Binding	Metabolic process	Organelle
Transporter activity	Localization	Protein-containing complex
Molecular function regulator	Biologic regulation	Extracellular region
Structural molecule activity	Multicellular organismal process	Membrane
Transcription regulator activity	Response to stimulus Biologic adhesion Developmental process

**Table 3:** Biologic pathway analysis

Neurologic pathways	Metabolism/nucleotide synthesis	Inflammation	Other signaling pathways
• Heterotrimeric G-protein Signaling Pathway-Gg alpha and Go alpha Mediated Pathway • Huntington Disease • Heterotrimeric G-protein Signaling Pathway-Gi alphal & Gs alpha Mediated Pathway • Ionotropic Glutamate Receptor Pathway • Metabotropic Glutamate Receptor Group I Pathway • Metabotropic Glutamate Receptor Group III Pathway • Muscarinic Acetylcholine Receptor 1 & 3 Signaling Pathway • Nicotine Pharmacodynamics Pathway • Nicotinic Acetylcholine Receptor Signaling Pathway • Parkinson Disease • Alzheimer Disease-Presnilin Pathway • Axon Guidance Mediated Semaphorins • Dopamine Receptor-mediated Signaling Pathway • Endogenous Cannabinoid Signaling	• Fructose Galactose Metabolism • Glycolysis • Pentose Phosphate Pathway • Pyrmidine Metabolism • Denovo Purine Biosynthesis • Denovo Pyrimidine Deoxyribonucleotide Biosynthesis	• Inflammation-mediated by Chemokine/Cytokine Signaling Pathway • Histamine H1 Receptor Mediated Signaling Pathway • B cell activation	• Wnt Signaling Pathway • Angiotensin II-stimulated Signaling *via* G proteins & Beta-arrestin • Integrin Signaling Pathway • PDGF Signaling Pathway • Ras Pathway • TGF-Beta Signaling Pathway • Apoptosis • Gonadotropin-releasing Hormone Receptor Pathway • Cadherin Signaling Pathway • Endothelin Signaling Pathway • FAS Signaling Pathway

To determine if any conformational changes in cardiac giant AnkG may occur with reported human variants, we searched the SMART database to identify the giant AnkG protein domain patterns (22). We were searching for known and predicted structural domains, but unfortunately were unable to find any changes in the long tail of the large isoform, as there is no known three-dimensional or secondary structure information available. This underscores the importance of further study on cardiovascular structure and function of this potentially impactful protein.

## Discussion

Ankyrins are intracellular adapter proteins that function in structural support and, in the case of cardiovascular tissue, provide a scaffolding important for normal electrophysiologic function (23). As stated in the introduction, canonical AnkB and AnkG have clear demonstrable roles in cardiovascular function, the latter of which operates in concert with the Na_v_ channel (6, 9). Giant ankyrin isoforms, on the other hand, are less well understood in the heart.

We created a mouse model with a cardio-selective deletion of giant AnkG expression heart to investigate the cardiovascular roles of giant AnkG. For the first time, we identified that giant AnkG is present in the heart. Previous work suggests that giant AnkG may not be present and/or function in the heart, (10). Our new data supports that giant AnkG is expressed in the heart, albeit at significantly lower levels than canonical AnkG, particularly in adult heart. In the study by Jenkins *et al.*, (10) giant AnkG was evaluated in neurologic tissue, where although it was found to be expressed in adult animals, it was demonstrated exponentially higher in the p0 brain. Our data provides additional evidence that giant AnkG is likely most important in early development, as it was more highly expressed in the neonatal myocardium.

Both structural and electrophysiologic roles for giant AnkG were elucidated in our study. From a structural standpoint, it is interesting that chamber dilatation by echo appeared mild, yet heart weight was significantly increased in cKO animals. However, one of the main differences that occurs in concentric *versus* eccentric hypertrophy is that in the former there is increase in myocyte width, but in the latter increases in both width and length are demonstrated. Human studies evaluating heart weight in these subtypes suggest that eccentric hypertrophy may result in a heavier heart due to these changes (24), similar to what is seen in cKO here. In giant AnkG cKO animals, atrioventricular block and ventricular arrhythmia upon adrenergic stimulation were identified, supporting a role for giant AnkG in stabilization of the myocardium. It is not surprising that ventricular arrhythmia was more common in cKO animals, given that we demonstrated structural abnormalities including fibrosis, which is known to be arrhythmogenic. Interestingly, in humans, high-degree atrioventricular conduction abnormalities are not common in DCM or similar phenotypes, and to our knowledge this finding is unique to the giant AnkG cKO animals. We hypothesize that the early developmental presence of giant AnkG in the heart may be necessary for normal conduction system development. Therefore, attenuated or aberrant giant AnkG function (either in the cKO animal or due to a giant AnkG mutant) may predispose to inherent developmental damage in the structure of the cardiac conduction system and result in defective conduction. Animals with cardiac-restricted giant AnkG deficiency in our experiments demonstrated eccentric hypertrophy with mild systolic dysfunction *and* evidence of abnormal electrical function. However, our single myocyte studies that illustrate altered APD phenotypes and myocyte contractility suggest that giant AnkG may be playing additional roles in intracellular and potentially calcium-based pathways. Likely, this is most pronounced in the developing animal.

An alternative but more likely hypothesis is that giant AnkG, unlike canonical AnkG, has additional functional roles completely independent of the sodium channel. EADs and DADs in the presence of normal *I*_Na_ suggest that intracellular calcium levels may be abnormal, and our myocyte studies support this, as cKO myocytes were more contracted at baseline and exhibited a greater percent peak shortening accompanied by normal relaxation time. Our computational data suggests that peak CSQN (a surrogate for DAD-inducing potential) is affected by increases in Ca^2+^ uptake or alternatively decreases in Ca^2+^ release. This may suggest that giant AnkG in the heart has functional roles in maintaining physiologic levels of Ca^2+^. This may occur through traditional effects *via* the L-type Ca^2+^ channel or ryanodine receptor or from alteration in reuptake *via* SERCA or NCX (25, 26). In addition, recent evidence suggests that store operated calcium entry (SOCE) pathways are likely more important in cardiomyocytes than previously thought (27, 28) and may be particularly important in neonatal cardiomyocytes (29). Our data suggests that giant AnkG is important in early life for normal heart function. It will be important in future experiments to evaluate the role of giant AnkG and Ca^2+^-based machinery in the developing heart.

Our transcriptional analysis supports overlap in the biologic pathways important for giant AnkG function in neurologic and cardiovascular tissue. In this analysis, we sought to determine if there were distinct, previously unstudied pathways, which might offer alternative hypotheses to the structural and electrical changes. Our analysis uncovered potential metabolic and inflammatory pathways, in addition to neurologic pathways, which was not surprising, given the documented role of giant AnkG in neurologic tissue (10). This is important to consider, because there is evidence for human neurologic disease as a result of mutations in giant AnkG. For instance, a human giant AnkG variant (p. Pro2490Lys) has been implicated in neurodevelopmental disease (30). In animals, mutations such as p. (Pro2490Lys) resulted in abnormal AIS assembly characterized by inability to achieve late developmental conformational change to the necessary extended protein form. Functionally, this mutation resulted in impairment of recruitment of βIV spectrin to the AIS and copattern of the giant AnkG partner neurofascin and VGSC. Given the evidence of roles for giant AnkG in human neurologic disease, areas of future study may include evaluating potential roles in cardiovascular physiology and disease.

In summary, giant AnkG is present in the heart and is enriched in 1-week-old mice, suggesting that it functions in important roles during early development. Mice with cardiac-specific deficiency of giant AnkG displayed eccentric hypertrophy, chamber thinning, and mild systolic dysfunction, consistent with a DCM phenotype characterized by structural changes and aberrant electrical conduction and enhanced arrhythmogenicity. The presence of DADs and EADs in the setting of normal *I*_*Na*_ supports the hypothesis that the giant AnkG isoform can function independent of the sodium channel in the heart, and our myocyte and computational data support that mechanisms responsible for increased Ca^2+^ uptake and/or decreased Ca^2+^ release will need to be the topic of future studies.

## Experimental procedures

### Animals

Mice with flanked exon 37 by loxP sites were crossed with αMHC-Cre knock in mice to produce animals specifically deficient in giant ankyrin-G exon 37 in the heart (*Ank3* e37^f/f^ x α MHC-Cre; “giant: AnkG cKO”) (10). *Ank3* e37^f/f^ floxed age- and sex-matched littermates were used as controls. Floxed wild-type (control) and giant AnkG cKO mice were studied in neonates (day of life 7) and both young (starting at 10 weeks) and aged (54 weeks) cohorts. All animal studies and surgeries were performed in accordance with the American Physiological Society Guiding Principles for Research Involving Animals and Human Beings and approved by the Institutional Animal Care and Use Committee at The Ohio State University.

### Immunoblot

Quantitated 30 μg (RCDC assay, BioRad) 22-week murine whole-heart lysates (in SDS/BME lysis buffer (10 mM sodium phosphate buffer pH 6.8, 2 mM EDTA, 10 mM NaN_3_, 12 mM NaCl, 1% SDS, 1% BME)) were loaded into 3 to 8% precast gels (NuPAGE) and transferred to Tris-Acetate protein membranes, which were blocked for >1 h at room temperature in 5% milk and incubated in primary antibody overnight at 4 °C. We used the giant ankyrin-G antibody at a concentration of 1:1000 (Gift Paul Jenkins Laboratory) and Goat-anti-rabbit 1:10,000 (Jackson Laboratories) for secondary antibody (10). For control, we utilized densitometric quantification (Image Lab, Version 5.2.1 build 11.2014.) and normalized to total protein content to calculate the relative intensity of the giant AnkG isoform. For 4-week-old mice, protein expression data, 100 μg of cardiac lysates, and 10 μg of brain lysate were used to check for the expression of giant AnkG (1:750, a gift from Dr Paul Jenkins), canonical AnkG (1:1000, a gift fro m Dr Paul Jenkins), and alpha-actinin (1:1000, Millipore Sigma A7811), was used as a loading control (10). For PCR, the following AnkG primers were used: giant AnkG (480 kDa): F: 5′-GAGGCACCGCCCTTAAA-3′, R: 5′-GCCAGCTCTGTCCAACTAA-3′ and for canonical AnkG (190 kDa): F: 5′- GCTTTGCCTCCCTAGCTTTA-3′, R: 5′- GATATCCGTCCGCTCACAAG-3′.

### Telemetry

Mice were implanted with ETA-F10 radiotelemeters (Data Sciences International; DSI PhysioTel). Continuous 2-h telemetry monitored recordings were performed to characterize the average resting HR 2 weeks after telemeter implantation. All ECG and telemetry tracings were analyzed with Labchart, ADinstruments (v.8).

### Echocardiography

From 10 to 54 weeks of age, littermate and cKO animals underwent bimonthly noninvasive transthoracic echocardiography (GE Logiq e) with the L10-22 (mHz) transducer. Mice were anesthetized using 2.5% isoflurane in 95% O_2_ and 5% CO_2_. Anesthesia was maintained by administration of oxygen and approximately 1.25% isoflurane during the whole imaging procedure. HR was monitored throughout imaging and recordings taken at a HR less than 400bpm were excluded from the analysis. We obtained parasternal long-axis images inclusive of two-dimensional loops and freeze-frame end-diastolic images to measure septal thickness, end-diastolic LV cavity dimension (LVID, d), and posterior wall thickness (LVPW, d). Additionally, we collected parasternal short-axis images at the level of the papillary muscles, inclusive of M-mode imaging, to determine the LV anterior wall dimension (LVAW), posterior wall (LVPW), and LVID in both systole and diastole. Functional data was derived from both parasternal long-axis two-dimensional real-time 3-beat loops and M-mode measurements, including fractional shortening (FS%) and ejection fraction (EF%).

### Electrophysiology

Whole-cell voltage- and current-clamp recordings were carried out in isolated adult mouse ventricular myocytes using standard patch-clamp techniques. Recordings were done using the Axopatch 200B amplifier (Molecular Devices). Sodium currents (*I*_Na_) were recorded at room temperature (20–22 °C) with pipette resistances of <2.0 MΩ when filled with pipette filling solution containing (in mM) five NaCl, 135 CsF (135), ten EGTA, five MgATP, and five HEPES at pH 7.2. The extracellular bathing solution contained (in mM) 5 NaCl, 1 MgCl_2_, 1.8 CaCl_2_, and 0.1 CdCl_2_. To assess the *I*_Na_ density, cells were held at −160 mV and stepped to various test potentials from −100 to 10 mV in 5-mV increments, with 200-ms duration pulses and 2800-ms interpulse intervals. Peak *I*_Na_ was assessed by an algorithm that calculates peak inward signal. As a result, any outward *I*_Na_ returns a zero value. Voltage dependence of inactivation was assessed by holding the cells from −140 to −40 mV for 300 ms, followed by a 30-ms test pulse to −40 mV; interpulse interval was 2700 ms. Recovery from inactivation was studied by holding cells at −160 mV and applying two 20-ms test pulses (S1, S2) to −45 mV, separated by increasing increments of 1 ms to a maximum S1-S2 interval of 50 ms. The S1-S1 interval was kept constant at 2000 ms.

Action potentials were elicited using square wave pulses (1–2 nA amplitude, 1–2-ms duration), generated by a Axopatch 200B amplifier (Molecular Devices), and recorded at room temp with pipette solution containing (in mM) one MgCl_2_, one EGTA, 150 KCl, five HEPES, five phosphocreatine, 4.46 K_2_ATP, 2 *b*-hydroxybutyric acid, adjusted to pH 7.2 with KOH, and extracellular solution containing (in mM) 148 NaCl, 0.4 NaH_2_PO_4_, one MgCl_2_, 5.5 glucose, 5.4 KCl, one CaCl_2_, 15 HEPES, and one EGTA, adjusted to pH 7.2 with NaOH. Appropriate corrections for access resistance compensation, as well as for leak and capacitive current subtractions, were made.

### Neonatal myocyte isolation and analyses

Myocytes were isolated using a Langendorff-free method published in detail elsewhere, with slight modifications for 1-week-old mice (31). In brief, enzyme concentration was reduced by 10% and Protease XIV was omitted. Buffers were adjusted to pH 7.8 using NaOH. Post isolation and filtration, CaCl_2_ was used to increase the calcium concentration stepwise with four additions in 10 min intervals, before spinning and resuspension in culture media for a final concentration of 1 mM CaCl_2_. Cardiac myocyte function was recorded using an Ion-Optix system using real-time sarcomere analysis. For intracellular calcium measurements, cells were loaded with 1 μg/ml Fura 2-AM for 20 min in culture media, then placed in culture media without FURA for 20 min before stimulation. Cells were stimulated in contractile buffer at 2 mM CaCl_2_ concentration using the Ion-Optix MyoPacer Field Stimulator equipped with a STIM-AT electrode (Cell Microcontrols) in stimulation chambers at 1 Hz for data collection. Collected cell data were then averaged and plotted using Graphpad Prism.

### Computational biology and modeling

Computer simulations were performed to predict mechanism for delayed after depolarizations (DADs) in the absence of overt changes to baseline action potential. The Bondarenko murine ventricular myocyte cell model (16) in the LongQt platform (32) was used for all simulations. In the Bondarenko model, DADs are triggered when concentration of Ca^2+^-bound calsequestrin (peak CSQN) in the sarcoplasmic reticulum (SR) reaches 11.5 (mM). Therefore, peak CSQN was used as a surrogate for DADs throughout these studies. Partial least-squares regression analysis was performed, as previously described (33), to determine relative impact of major ion channels and transporters in the model on peak CSQN and action potential amplitude, duration at 75% repolarization (APD_75_), and max derivative. A total of 200 single-cell simulations were performed with random perturbation (log normal distribution, with a mean of 0 and a standard deviation of 1.0) of scaling parameters for the following ion fluxes: L-type Ca^2+^ current (g_Ca(L)_), Na^+^/Ca^2+^ exchanger (g_NaCa_), Na^+^/K^+^ ATPase (g_Nak_), sarcolemmal Ca^2+^ pump (g_Ca(P)_), Ca^2+^ leak flux from the SR (g_leak_), Ca^2+^ release from SR (g_rel_), Ca^2+^ translocation from network to junctional SR (g_tr_), Ca^2+^ flux to troponin (g_trpn_), Ca^2+^ uptake into SR (g_up_), and Ca^2+^ flux from the subspace volume to bulk myoplasm (gxfer). Following regression analysis, simulations were performed where a subset of the parameters were incrementally increased (0.2 steps starting at 0.1–2.9) and evaluated for their effect on peak CSQN, action potential amplitude, APD_75_, and max derivative. The cell model was paced using a stimulus of −60 mV with a 0.5 ms duration for 30,000 ms at 0.5 Hz, followed by an increase in pacing frequency every 10,000 ms to 1,2,5 and finally 10 Hz, after which pacing was abruptly stopped and the cell was allowed to return to baseline for 50,000 ms.

### Transcript analysis

We collected whole heart specimens from cKO and floxed-control adult animals at 22 weeks of age to include: three male cKO, three male control, three female cKO, and three female control samples. Gene microarray analysis was conducted as previously described (34). Total RNA was isolated using TRI Reagent (Molecular Research Center, Part # TR118). After isolation, RNA was quantified and treated with RNase free DNase I (Epicentre; Part # D9905K) and repurified using RNA Clean XP magnetic beats (Beckman Coulter Part # A63987). Amplified cDNA libraries were prepared from 200 ng of DNA-free total RNA using the Universal Plus mRNA-Seq Library Prep Kit (NuGEN Technologies, Inc; Part #0509-96). Chip-based capillary electrophoresis on Agilent 2100 Bioanalyzer High Sensitivity DNA assays (Agilent Technologies; Part # 5067-4626) was used to analyze quality of the libraries, which were then quantified with the Takara Library Quantification Kit (Part # 638324).

### Quantitative real-time PCR

Real-time PCR was performed as previously described (8). Briefly, Total RNA from the mouse heart tissues was extracted with TRIzol Reagent (Invitrogen) following manufacturer's instructions; 1 μg of total RNA, treated with ezDNase, was used for the first-strand complementary DNA synthesis using SuperScript IV Vilo Master Mix (Thermo Fisher Scientific). qRT–PCR reactions were performed in triplicate in 96-well optical plate with PowerUp SYBR Green Master Mix (Thermo Fisher Scientific). Primers for Giant AnkG (480 kDa) F: 5′-GAGGCACCGCCCTTAAA-3′ and R: 5′-GCCAGCTCTGTCCAACTAA-3′ (10) *HPRT* levels were used as a normalization control.

### Statistics

Continuous variables were analyzed with unpaired Student’s *t*-tests (two-tailed) when evaluating single comparisons and with one-way analysis of variance in the case of multiple comparisons. Categorical variables were evaluated with Chi-Square and Shapiro–Wilks criteria were used to assess the distribution of data and whether or not nonparametric testing was indicated. An α level of <0.05 was considered statistically significant and all analyses were performed using JMP Pro, Version 12.2.0. SAS Institute Inc, 2015.

## Data availability

All data is contained within the article and supporting information document.

## Supporting information

This article contains supporting information.

## Conflict of interest

The authors declare that they have no conflicts of interest with the contents of this article.
