# Supplementary material for: Giant ankyrin-G regulates cardiac function
Source: J Biol Chem. 2021 Mar 4;296:100507. doi: 10.1016/j.jbc.2021.100507 (PMC8040283; doi:10.1016/j.jbc.2021.100507)
Supplement: Supplemental Figures S1 and S2 [file mmc1.docx]

**Supporting Information**

**Giant ankyrin-G regulates cardiac function**

Omer Cavus MD^1,2^, Jordan Williams BS^1,2^, Hassan Musa PhD^1,2^, Mona El Refaey PhD^1,2^, Dan Gratz BS^2,3^, Rebecca Shaheen BS^2,3^, Neill A. Schwieterman BS^1,2^, Sara Koenig PhD^1,2^, Steve Antwi-Boasiako BA^2^, Lindsay J. Young BS^1,2^, Xianyao Xu MS^1,2^, Mei Han, MD^1,2^, Loren E. Wold PhD^1,2^, Thomas Hund PhD^2,3,4^, Peter J. Mohler PhD^1,2,4^, Elisa A. Bradley MD^2,4^

^1^ Department of Physiology and Cell Biology, The Ohio State University, Columbus OH

^2^ The Frick Center for Heart Failure and Arrhythmia, Dorothy M. Davis Heart and Lung Research Institute, The Ohio State University, Columbus OH

^3^ Department of Biomedical Engineering, College of Engineering, The Ohio State University, Columbus, OH

^4^ The Ohio State University Department of Internal Medicine/Division of Cardiovascular Medicine, Columbus OH

**Figure S1. Confirmation of Giant AnkG Deletion**


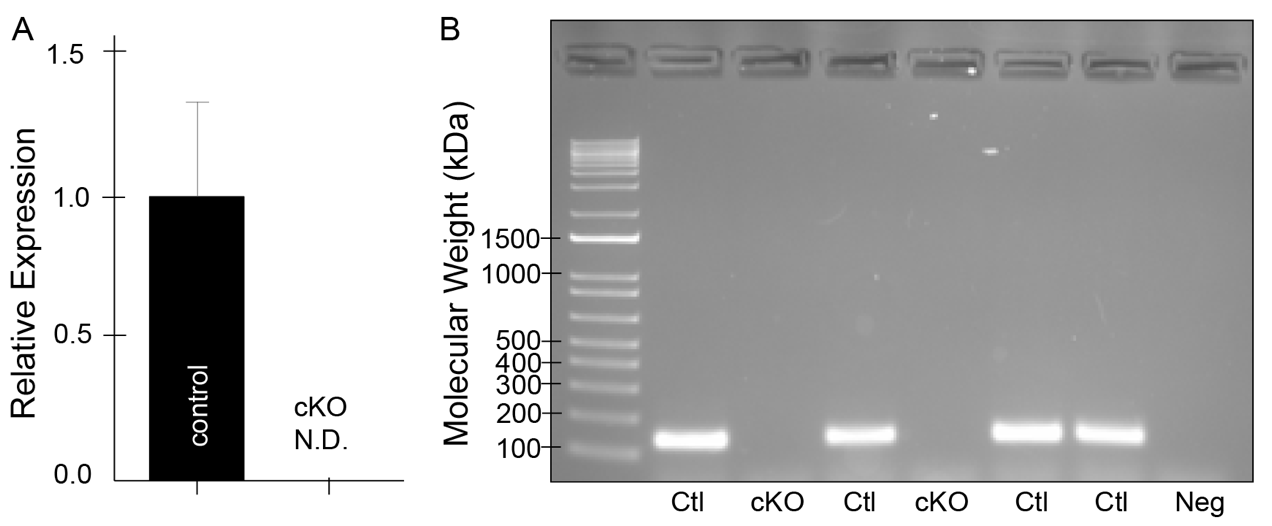


Figure S1. Relative expression (real time PCR) of giant AnkG in 4 week old mice demonstrated no detection (N.D.) in the cKO (n = 2) as compared to floxed control animals (1.0 + 0.3, n=4) (**A**). To confirm that giant AnkG was selectively silenced, PCR products were run on gel, and demonstrated no evidence of giant AnkG in cKO animals (**B**).

**Figure S2. Resting electrocardiographic findings in Giant ankyrin-G cKO and control mice**

**
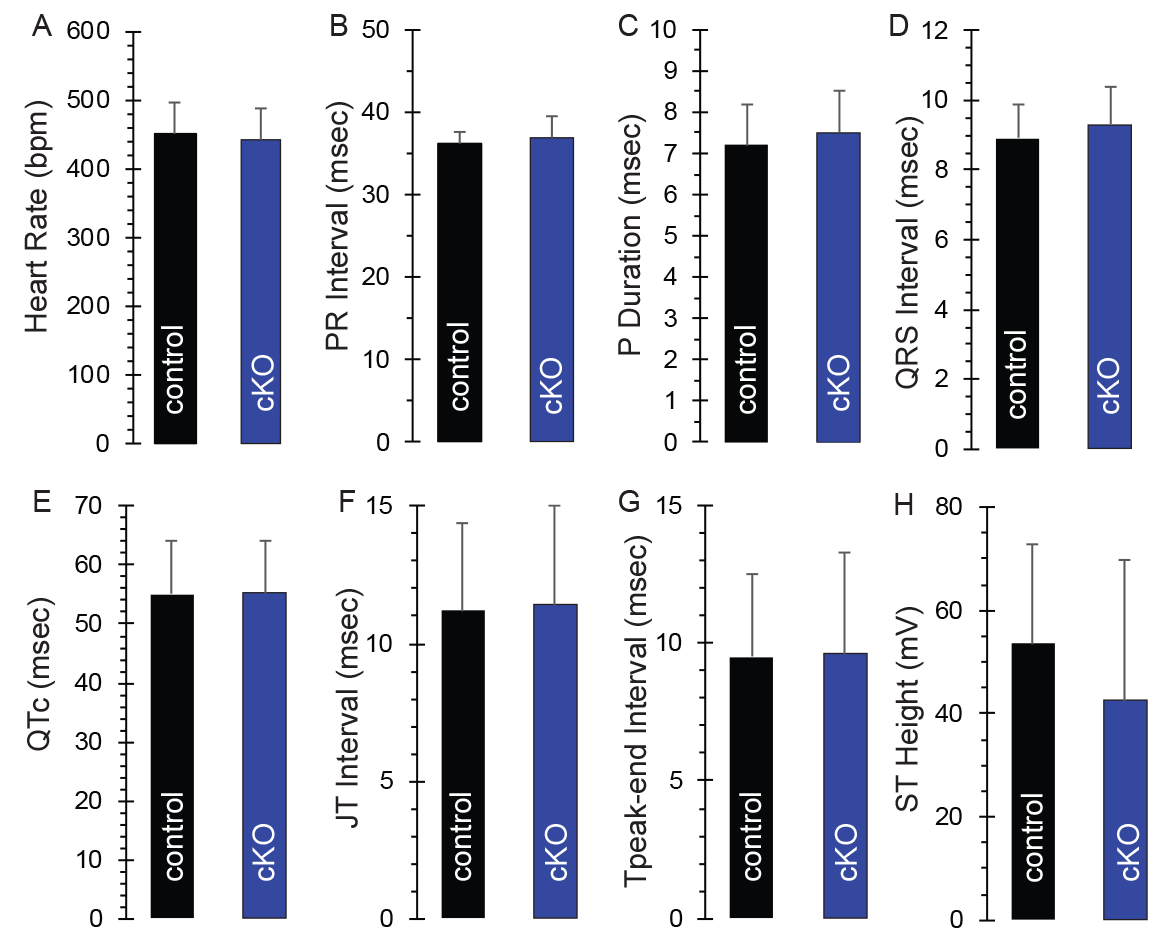
**

Figure S2. Giant AnkG cKO mice demonstrated no significant abnormalities in resting electrocardiographic parameters including intervals, duration, corrected intervals and amplitudes. The included parameters are collected during resting ECG according to the study protocol described. In addition, awake heart rates were recorded and found to be no different between control and cKO mice (735 + 31 vs. 757 + 31bpm, p = 0.08) (Control: n = 14, cKO: n = 16).
